# Implementation by simulation; strategies for ultrasound screening for hip dysplasia in the Netherlands

**DOI:** 10.1186/1472-6963-10-75

**Published:** 2010-03-23

**Authors:** Sabrina Ramwadhdoebe, Godefridus G Van Merode, Magda M Boere-Boonekamp, Ralph JB Sakkers, Erik Buskens

**Affiliations:** 1 Department Orthopaedics, University Medical Center Utrecht, Utrecht, Netherlands; 2 Faculty of health, medicine and life sciences, Maastricht University Medical Center, Maastricht, Netherlands; 3 Department of Science, Technology, Health, and Policy Studies (SteHPS), School of Management and Governance, University of Twente, Enschede, Netherlands; 4 Julius Center for health sciences and primary care, University Medical Center Utrecht, Utrecht, Netherlands; 5 Medical Technology Assessment, Department of Epidemiology, University Medical Center Groningen, University of Groningen, Groningen, Netherlands

## Abstract

**Background:**

Implementation of medical interventions may vary with organization and available capacity. The influence of this source of variability on the cost-effectiveness can be evaluated by computer simulation following a carefully designed experimental design. We used this approach as part of a national implementation study of ultrasonographic infant screening for developmental dysplasia of the hip (DDH).

**Methods:**

First, workflow and performance of the current screening program (physical examination) was analyzed. Then, experimental variables, i.e., relevant entities in the workflow of screening, were defined with varying levels to describe alternative implementation models. To determine the relevant levels literature and interviews among professional stakeholders are used. Finally, cost-effectiveness ratios (inclusive of sensitivity analyses) for the range of implementation scenarios were calculated.

**Results:**

The four experimental variables for implementation were: 1) location of the consultation, 2) integrated with regular consultation or not, 3) number of ultrasound machines and 4) discipline of the screener. With respective numbers of levels of 3,2,3,4 in total 72 possible scenarios were identified. In our model experimental variables related to the number of available ultrasound machines and the necessity of an extra consultation influenced the cost-effectiveness most.

**Conclusions:**

Better information comes available for choosing optimised implementation strategies where organizational and capacity variables are important using the combination of simulation models and an experimental design. Information to determine the levels of experimental variables can be extracted from the literature or directly from experts.

## Background

Implementing new technologies in organizations may not be straightforward and simple. Not only will new technology affect the organization itself but many times the external environment of the organization as well[1]. Nowadays, also the economics of implementation as such are being considered more and more [2-4]. In practice, however, only one or two implementation strategies a priori considered feasible by the stakeholders are taken into account for (economic) evaluation. This limitation is largely due to financial and practical constraints to analyze more strategies. Mostly this implies leaving a number of potentially interesting and viable scenarios unexplored.

To prepare for a wider implementation of a new screening modality regarding developmental dysplasia of the hip (DDH) in Dutch infant health care centers (IHC) we will first evaluate different implementation strategies. Earlier studies have shown that the current screening strategy based on physical examinations and identification of known risk factors leads to less favorable outcomes and waste of scarce health care resources compared to ultrasound (US) screening. On the other hand, reviews have also shown that there is doubt regarding the effectiveness since over treatment may occur [5,6]. Implementing US screening is expected to lead to lower referral rates to specialists. The Dutch Soundchec 1 study showed that US screening would be most (cost-) effective at the age of three months[7]. The Soundchec 1 study assessed which strategy was (cost-) effective compared to current screening. The strategies that were studied were selective screening, i.e., only children with risk factors for DDH, and general ultrasound screening at the age of one, two and three months. Based on this result, an implementation study referred to as the Soundchec 2 study, was launched to find and solve problems associated with future nationwide introduction of US screening. In total 4600 children will be invited for US screening for an implementation study in two different regions[8]. In order to facilitate national implementation of the screening program within the current health care system information on possible risks, benefits, quality and costs of US screening for detecting DDH should be assessed.

By modeling a number of implementation strategies we expected to identify potentially viable implementation strategies, which may subsequently be subjected to more elaborate experimentation. Our ultimate objective was to explore various implementation models for US screening in the current system and simultaneously estimate the cost-effectiveness thereof. Using an experimental design we explicitly assessed how input variables affect the outcome such as cost-effectiveness[9]. To see which of the scenarios presented in the experimental design performed best, a system analysis can be conducted using a simulation model[9].

## Methods

We used an experimental design with the subsequent steps presented in Additional file 1: Table S1 [10]. We began by analyzing the existing workflow and then defined the relevant performance measures. Next alternative workflow scenarios were conceived during focus group interviews. The software used to actually build the model was ARENA[11]. Finally, through experimentation with the alternative scenarios, those yielding optimal performance could be identified. A societal perspective was used for the analysis which means that costs for all potential payers are considered. Thus using the simulation model we were able to estimate the expected effects of all scenarios and it became clear which factors influenced the outcomes most. Based on the results from the simulation model the best performing alternatives may be selected, or possibly new further improved scenarios may be drawn up. The overall results may be used directly by policy makers or can be used in additional implementations studies. The examination protocol of the Soundchec 2 study was reviewed by the Medical Ethical Boards which judged that approval was not necessary because no experimental tests subject to the law on medical scientific research were applied on humans.

## Results

### Step 1. Define performance measures

The variation between scenarios was reflected in the variation of the cost per screen detected child which was calculated for each scenario. A societal perspective was used for the analysis which means that costs for all potential payers are considered.

Costs for diagnosis, referral, treatment of dysplasia of the hip, and time costs for the parents were included. The relevant clinical outcome was defined as a child detected and treated with treatment occurring within a year after diagnosis (true positives of screening). In essence, we summed and compared all the costs incurred for the screening and treatment according to the various scenarios and divided these by additional number of children detected and treated. We also included the cost of the missed cases and false positives.

### Step 2 and 3. Analyze the performance of the existing workflow: 'Current and new strategy for detecting DDH'

In the Netherlands the current strategy for detecting children with DDH consists of history taking and physical examination during routine consultation at the infant health care centers (IHC) organized by youth health care organizations. Parents visit the IHC at regular intervals and consultation either the nurse or the physician. At the age of 1, 3, 6, 9 and 14 months the consultation with the physician comprises identification of risk factors and a physical examination of the hips. In case of a positive test result, the child is referred to a general practioner (GP). The GP will repeat the tests performed by the IHC physician and will generally refer the child to the radiology department or an orthopedic surgeon. In an outpatient setting an ultrasound (US) and/or X-ray and an orthopedic examination are performed to determine whether DDH is present. In almost 84% of the children referred the presence of DDH is refuted based on these specialist exams[7,12].

The new screening strategy requires that US examinations of the infants' hips are performed on the hips at the age of three months. According to the principles of Graf hips can be classified in six classes (type 1, 2b, 2c, D, 3 and 4) where type 1 is normal and type 4 complete luxation of the hip[13]. IHC-nurses, IHC physicians and radiographic technicians (RT) who work at the IHC may potentially qualify as screeners. These disciplines were trained to perform the examinations. However, the selection of disciplines performing the US examination may influence the cost-effectiveness in two ways. If physicians should appear better at performing the examinations this implies increased costs in terms of wages but on the other hand this might imply higher effectiveness. Also, at an organizational level the cost-effectiveness attained may be influenced by the available capacity of screening personnel. This also applies to actual rooms and equipment required to perform the US examinations, training capacity, planning etc.

Regardless of the screening strategy there will be children who have a higher risk of DDH. In the current screening, all children whose parents during history taking state that one of the known risk factors is present are sent to their GP and/or an orthopedic surgeon regardless of the results of the physical examination. In the situation where US screening is implemented, we presumed that only those with a positive screening result would be referred to a medical specialist, i.e., instead of acting on possible risk factors being present. In table 1 the values in terms of missed cases, false positives and current screening and US-screening are given.

**Table 1:** Performance measures of the current screening and the US-screening at the age of three months.

Item	Current screening	US-screening
Missed cases	0.9%	0.6%

False positives	16.5%	1.3%

True positives	2.8%	3.2%

Percentages reflect the number of missed cases, false positives and true positives as proportion of the total number of screened children.

Source [7]

### Step 4 and 5. Brainstorm about improvement, define alternatives

By exploring changes in cost-effectiveness ratios we could actually explore whether alternatives values of the experimental variables (input) might be defined in such a way that these remained plausible. The next step was defining alternative implementation scenarios. We used the ideas and comments which had been stated in focus group interviews among stakeholders such as policymakers, managers in the IHC and screeners (physicians, nurses and radiographic technicians). The choices for these groups were based on the evaluation of the Soundchec 2 project team. For each of these groups of professionals separate interviews took place. In each group four to eight persons participated. The purpose for the interviews was to discuss possible limitations and opportunities for the implementation of ultrasound screening. These interviews provided important insight regarding the definition of the experimental variables, and also for performance measures. The complete article describing the focus group interviews will be published by the project team at a later phase.

Important performance measures defined were the attendance rate and the quality of screening A high attendance is critical for effective screening[14]. This performance measure will provide important information for the policy makers and the IHCs. Attendance in rural areas may be different from that in urban areas[15]. In rural areas people need to travel more because of the spread of IHCs, which likely influences attendance. On the other hand, in urban areas more people who have different cultural background reside, which may again influence the attendance rate. Another important aspect of screening which can influence the attendance rate is the organizer of the screening. The interviewees suggested that screening should be planned and conducted by an easy access organization which is familiar to parents. The quality of the screening needs to be compared with the current screening strategy. In a previous study (Soundchec 1) the rates of missed cases, treated and false positives were established[7]. Quality of screening is an important outcome measure since missed cases and false positives will influence the cost-effectiveness.

Ideas that were brought forward for defining the experimental variables were:

- availability of US-machines at every IHC

- implementation of the screening outside the IHC- organizations

- consultation in evening hours

- implementation of the screening combined with routine three month consultation, this would imply at every IHC an US machine

- guaranteed high quality screening

- premise to call it screening is a high attendance, which may be achieved through organization of the screening close to the residence of parents

- influence of travel distance

- no purchase of US machines, but make use of available US machines in other settings, e.g. in obstetric centers.

From these global ideas four experimental variables were derived which were considered to be pivotal for the organization of ultrasound screening at IHCs. These variables were subsequently used for experimentation (table 2).

**Table 2:** Experimental variables

Experimental variables	
A. Number of portable US machines	1. As many as necessary US machines 2. Limited number of US machines 3. Externally organized (no US machines purchased)

B. Consultation	1. Integrated consultation with regular consultation at the age of three months 2. Extra consultation

C. Screener	1. Infant health care physician 2. Infant health care nurse 3. Radiographic technician (RT) 4. Medical specialist (radiologist or orthopedic surgeon)

D. Location and time	1. Daytime at IHC 2. Organized in buildings not owned by IHC (external) 3. Organized in evening hours at IHC.

Based on these experimental variables, 72 possible scenarios can be drawn up. With the following assumptions, all scenarios were run in a simulation model:

1. If the screeners are employees currently working in the IHC and/or the location of the screening is in buildings owned by the IHC, the screening is organized by the IHC.

2. An integrated consultation implies a regular three month consultation comprising an US examination. When the screener is a radiographic technician or a medical specialist, the infant health care physicians have to be present at the location of screening to perform the regular IHC consultation.

3. An integrated consultation with an infant health care nurse as screener, means that infant health care physicians will delegate their tasks in the regular three month consultation to the infant health care nurse. Delegation (substitution) of tasks under specified conditions is increasingly applied in IHC [16,17].

### Step 6. Experimentation with alternatives

#### Simulation model

The simulation model was build in ARENA[11]. Each scenario was evaluated using sequences where patients go to different stations defined by the sequence.

To model each of the 72 scenarios the levels for each of the experimental variables were set. The levels were set in accordance with expert opinion and data gathered from IHC organizations. Besides the levels of the experimental variables, the attendance rate and the quality of screening examinations were quantified. This was achieved using data available from IHC-organizations (attendance rate and cost price) and expert opinion (quality of screener). Other information needed to run the model was based on the reports of the Soundchec 1 study [7], Dutch guideline for cost-effectiveness studies [18], IHC-organization reports [15]and expert opinion.

The population simulated repeatedly comprised 2300 children expected to visit the IHC at regular time intervals over a period of 18 months. This figure corresponds with our ongoing implementation study. We excluded the children who were referred and treated in their first three months of life.

#### Quality and attendance rate

The quality of the screener is defined in terms of cases missed (false negatives) and false positives, both as a proportion of the total number of screened children. Since only the proportions were known for radiographic technicians (Soundchec 1 study), we set the proportions for IHC physicians, IHC nurses and medical specialists by expert opinion (Table 3).

**Table 3:** Quality of screening result for different screener type.

Experimental variable	False positives	Missed cases
Infant health care physician	1.4%	0.6%

Infant health care nurse	1.5%	0.7%

Radiographic Technician[19]	1.3%	0.6%

Medical specialist	0.6%	0.3%

Presented percentages reflect the number of missed cases or false positives as proportion of the total number of screened children.

Attendance rate was based on three participating organizations in the screening program. One organization in a rural area and two organizations in an urban/suburban area. For the rural area we used an attendance rate of 90% and for the urban area a rate of 85%. The re-attendance rate (after a reminder) for the urban area was estimated to be 80% and 90% for the rural area.

#### Cost items

With regard to the relevant estimates of cost items travel time is based on the Dutch guideline for CE analyses [18]: 0.20 per kilometer by car or public transportation. The travel distance to the different locations are 4-7 kilometers for IHC and ten for external locations[15]. Estimates of treatment costs were based on the Soundchec 1 study [7-18]. Treatment costs were included for each Graf type. Also the treatment cost of current screening after one and two months were based on this study. For this period, treatment costs for Graf type 2a were included since this type is only given to infants up to two months of age. The cost of false positives in current screening consisted of one hospital visit including the cost of absence of work of the parents (€61 + €36). In the model, the salaries were adjusted when screening was done in evening hours (35% extra costs) and when an extra consultation took place (double the salary costs and travel time).

For cost of training we assumed that also medical specialists would need training since many of them do not know how to perform US-screen for DDH and the setting is different. We further assumed two day training sessions lasting eight hours for the calculation. We used the average salary of the four screener types plus (16 hours * €73) an additional amount for salary of the instructors and material (€ 130). These numbers were justified by expert opinions who give training to radiographic technicians.

For the costs of depreciation of the machine we calculated the annuity for the total period of five years with an interest rate of 5% (€ 7560)[18]. Each machine costs € 32725, the insurance per year costs € 1000. We also included maintenance costs of 8% of € 32725 (€ 2618)[18]. The total costs per year are therefore € 11178, which resulted in an average of € 5 per child (2300 children). In table 4 all items are presented together with the point estimates and sources. Prices were adjusted using the price index rate (statline.cbs.nl) for the year 2006.

**Table 4:** Cost items, source and values

Item	Source	Value
IHC physician	IHC - CAO fwg 65	€ 75 per hour

IHC nurse	IHC - CAO fwg 45-50	€ 42 per hour

Radiographic technician	Hospital CAO	€ 70 per hour

Radiologist	Hospital CAO	€ 106 per hour

Ultrasound at hospital	Soundchec 1	€ 61 per hour

Treatment missed case	Average of type 2, D,3 and 4	€ 1217

Travel time	Dutch guideline for CE-analyses (price per kilometer)	€ 0.20

Training	Soundchec 2	€ 1300

Treatment	Soundchec 1	
Percentages:		
after first consultation at IHC.		- € 571
after US type 2b/c	1.4%	- € 897
after US type D	1.5%	- € 717
after US type 3/4	0.5%	- € 2043

Referral percentage first consultation	Soundchec 1	0.61%

Treated after first consultation IHC	Soundchec 1	0.31%

Time Parents	Dutch guideline for CE analyses	€ 36 per hour

Use US machine	Dutch guideline for CE studies	€ 5 per child

Overhead evening hours	35% of average salary of screeners	€ 5 euro per child

#### Explanation quantification levels of experimental variables

##### A. Number of Ultrasound machines

The scenarios for the levels pertaining to the number of US machines are defined in accordance with the following assumptions:

- Many machines: average number of IHC assumed to be efficient is seven based on literature and expert opinion [15],

- Limited: one US machine, which means one salary compartment and one overhead for each cycle.

- None: buildings will be used where US machines are already available, like in an obstetrics center or in a multilevel health care facility center.

##### B. Consultation

For one consultation at the IHC parents incur one hour productivity loss. For an extra consultation this would mean in total two hours of productivity loss for the diagnosis and detection of DDH. Extra travel time cost was also included for an extra consultation.

##### C. Screener

For each screener type the salary was calculated for ten minutes. For each screener, the variability of the quality of screening was included with the use of expert opinion.

##### D. Location

For each location attendance rate, travel time and costs were included. Extra cost for the evening hours was 35% of the average salary[18]. For an external location rent needed to be paid.

### Step 7. Select best alternatives

For each of the 72 scenarios we calculated the cost per screen detected child. For each scenario we divided the total cost by the number of true positives which gave us the cost effectiveness-ratio (CE-ratio). We divided the results in terms of iCERS in quartiles of cost-effectiveness (CE_A to CE_D), thus each group included 18 scenario's (see table 5). For each of the four groups we estimated the frequency of the experimental variables.

**Table 5:** CE-groups

Group	Cost-effectiveness range
CE_A low	3231 - 3469

CE_B moderate	3473 - 4188

CE_C intermediate	4190 - 4549

CE_D high	4794 - 5310

For experimental variable A we saw that the frequency for level 1 (many machines are bought) in the least cost-effective scenario's (group CE_ D) was high. For level 2 (limited machines are bought) we saw a slightly higher frequency in group CE_B and for level 3 (no machines are bought) a higher frequency in group CE_A.

#### Figure 1

**Figure 1:**
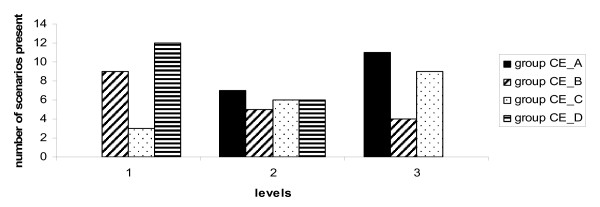
**Experimental variable A Number of ultrasound machines**. Quartiles of cost-effectiveness (CE_A to CE_D) ranging from most cost-effective (group CE_A) to least cost-effective (group CE_D). Levels used: 1. As many as necessary US machines, 2. Limited number of US machines and 3. Externally organized (no US machines purchased).

For Experimental variable B consultation the frequencies were mostly located in the most cost effective quartiles for level one (integrated consultation). Level 2 (extra consultation) was mostly located in the least cost effective quartiles.

#### Figure 2

**Figure 2:**
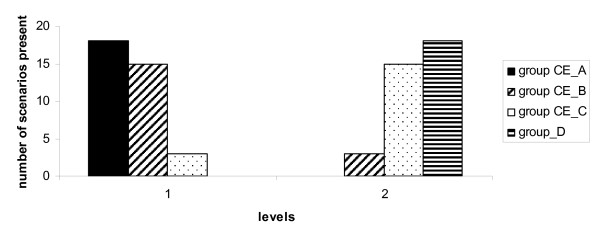
**Experimental variable B Consultation**. Quartiles of cost-effectiveness (CE_A to CE_D) ranging from most cost-effective (group CE_A) to least cost-effective (group CE_D). Levels used 1. Integrated consultation with regular consultation at the age of 3 months and 2. Extra consultation.

#### Figure 3

**Figure 3:**
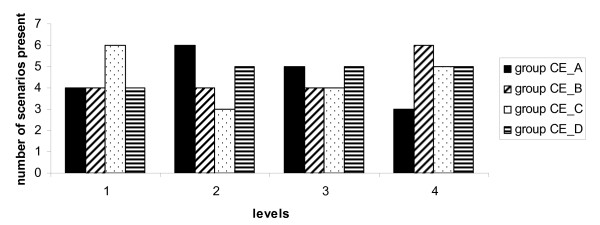
**Experimental variable C Screener**. Quartiles of cost-effectiveness (CE_A to CE_D) ranging from most cost-effective (group CE_A) to least cost-effective (group CE_D). Levels used 1. Infant health care physician, 2. Infant health care nurse, 3. Radiographic technician (RT) and 4. Medical specialist (radiologist or orthopedic surgeon).

For experimental variable C a less clear pattern could be revealed. The frequencies for level 1 (infant health care physicians) were high in group CE_C For level 2 (nurses) we saw a high frequency in group CE_A and for level 4 (medical specialist) a high frequency in group CE_B.

#### Figure 4

For level 1 (daytime in IHC centers) of experimental variable D we found that the frequency was highest in group CE_B and for level 2 (rented buildings) in group CE_C. Level 3 (evening sessions) was almost equally distributed among the four groups, but had the highest frequency in group CE_D.

**Figure 4:**
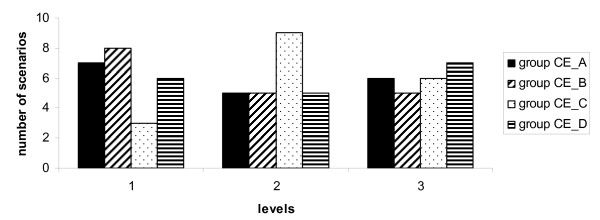
**Experimental variable D Location**. Quartiles of cost-effectiveness (CE_A to CE_D) ranging from most cost-effective (group CE_A0 to least cost-effective (group CE_D). Levels used 1. Daytime at IHC, 2. Organized in buildings not owned by IHC (external) and 3. Organized in evening hours at IHC.

#### Table 6 Least and most cost-effective scenarios

**Table 6:** Five Least en most cost-effective scenarios

scenarios	Experimental variable A Ultrasound machines	Experimental variable B Consultation	Experimental variable C Screenertype	Experimental variable D Location/time	Cost/Effect
47	3	1	2	1	3231

29	2	1	2	1	3273

55	3	1	4	1	3299

49	3	1	3	1	3321

64	3	1	2	3	3329

61	1	2	4	2	5159

15	1	2	2	2	5159

21	1	2	3	2	5249

69	1	2	4	3	5256

9	1	2	1	2	5310

The five most cost-effective scenarios (47, 29, 55, 49 and 64) and the least cost-effective scenarios (61, 15, 21, 69 and 9) are presented in table 6. The difference in cost per screen detected child between the least and the most cost-effective scenarios is approximately 2000 euro. This difference is due to the fact that in most cost-effective scenarios no US machines were bought (level 3 for experimental variable A) and screening took place in buildings currently owned by the IHC (level 1 experimental variable D). The screening is done by one of the four screener types and in three cases the screening takes is done by a nurse. All five cost-effective scenarios take place in an integrated consultation. For the least cost-effective scenarios we noted that there were many machines bought, the screening took place in a building that had to be rented sometimes or in the evening with additional salary costs and an extra consultation occurred.

## Discussion

Better information comes available for selecting optimised implementation strategies where organizational and capacity variables are important using the combination of simulation models and an experimental design. Information to determine the levels of experimental variables can be extracted from the literature or directly from experts. The variability and their interaction of independent variables on outcomes, for example cost-effectiveness, can be better revealed by analysing many possible strategies.

Early detection and treatment of DDH are important. Current screening methods using risk identification and physical examinations result in considerable numbers of missed cases and high proportions of false positives. Ultrasound screening of the infant hip was shown to be a cost-effective alternative screening method. In the Netherlands ultrasound screening may be organized in the current infant health care centers where children have regular consultations with physicians and nurses. How to best organize i.e., optimize screening for detecting DDH in terms of cost-effectiveness may not be revealed by choosing and implementing just one implementation strategy in an experimental setting. The use of a hypothetical experimental design in combination with a simulation model allows researchers to efficiently estimate the potential cost-effectiveness of the many possible implementation strategies or scenarios. Important information regarding the levels of the parameters in an experimental design may be derived from expert opinion and literature.

Four experimental variables with corresponding levels were set. In total 72 scenarios were evaluated using this design. The 4 experimental variables were defined for the number of US machines (A), the type of consultation (B), type of screener (C) and the location (D). The 72 cost-effectiveness ratios were divided in four groups, ranging from least cost-effective to most cost-effective. For experimental variable A we saw that for each level the highest frequency was located in a different cost-effectiveness quartile. This can be explained by the fact that for each additional US machine, training of additional screeners would be required. The levels of experimental variable B were right censored for level 1 and left censored for level 2. This means that an extra consultation was in the group for low cost-effectiveness and vice versa for level 2. This can be explained by the additional indirect costs that have to be made for an extra consultation (travel cost and absence of work). For experimental variable C Screener we see that the frequency for the different quartiles is different for each of the four levels. This can be explained by the fact that nurses and radiographic technicians may be less expensive but also perform less accurate. Medical specialists are more expensive but presumably more accurate. For the two expensive screener types (IHC-physician and medical specialist) the frequencies are the highest in group CE_C and CE_D. For nurses (level 2) the highest frequency is in group CE_A. For each of the levels of experimental variable D we saw that each level had the highest frequency in different groups in terms of cost-effectiveness. This can be explained by the difference in overhead costs or rent that for each location has to be paid. It is therefore straightforward that level one is located in the most cost-effectiveness groups CE_A and CE_B since no additional costs need to be made.

The five most cost-effective scenarios had in common that none included the level 'many US machines' and externally organised in buildings that have to be rented. On the other hand, all five included an integrated consultation. The five least CE-scenarios had in common that they all included the level many US machines, an extra consultation and organisation in evening times or external buildings. In our model the experimental variables related to the number of US machines in combination with an extra consultation affected cost-effectiveness the most.

With regard to the information used and the underlying assumptions we recognize that for nationwide implementation of screening the final parameter estimates may be different from those currently used. Also, to reflect uncertainty regarding point estimates (based on expert opinion), distributions around point estimates can be used. In this study the (number of children) detected and treated within a year were used for the outcome-analysis. When using cost-effectiveness analysis as primary outcome measure, QALY measurements should preferably be used. When using QALY as a measure of effect this facilitates comparisons between other cost-effectiveness analysis or maximum willingness to pay of a society. Because no other data were available, we used the data pertaining to the current screening strategy. The ongoing implementation study started by our study group will end in 2009. This study will yield accurate and within trial data on quality difference between the different types of screeners, attendance rate and the interaction effects between variables. The uncertainty with regard to the actual effectiveness of treatment of DDH has not been modeled. The cost for society of possible over treatment is in fact impossible to calculate since no randomized control trial has been done to evaluate the effectiveness of treatment on DDH. Children who are incorrectly referred (false positives) might receive treatment. This study assumes that false positives did not undergo treatment. No data are available on the number of children offered treatment after ultrasound screening. The simulation model described in this article was used to assess variation between scenarios not to estimate the exact cost-effectiveness of the scenarios.

## Conclusions

Further research is needed for the analyses of local capacities to optimally organize screening. These results will be presented later using a discrete-event simulation model where the competition for resources will be included. As an overall recommendation we believe that an experimental design and interviews with experts may restate and be used for the definition for alternative scenarios. The five candidate scenarios appear promising and should be subjected to further simulation and practical experimentation.

## Competing interests

The authors declare that they have no competing interests.

## Authors' contributions

SR carried out the analysis and drafted the manuscript

GGM participated in the design of the study, helped analysing the results and helped to draft the manuscript

MMB helped to draft the manuscript

RJBS helped to draft the manuscript

EB participated in the design of the study, helped analysing the results and helped to draft the manuscript

All authors read and approved the manuscript

## Pre-publication history

The pre-publication history for this paper can be accessed here:

http://www.biomedcentral.com/1472-6963/10/75/prepub

## Supplementary Material

Additional file 1 **Steps in experimental design**. A table showing 7 steps in an experimental design. Click here for file
