# Supplementary material for: Implementation by simulation; strategies for ultrasound screening for hip dysplasia in the Netherlands
Source: BMC Health Serv Res. 2010 Mar 23;10:75. doi: 10.1186/1472-6963-10-75 (PMC2851713; doi:10.1186/1472-6963-10-75)
Supplement: Additional file 1 — Steps in experimental design. A table showing 7 steps in an experimental design. [file 1472-6963-10-75-S1.DOC]

Table S1. Steps in experimental design

| 1. Define performance measures  2. Analyze the existing workflow  3. Determine the existing performance  4. Brainstorm about improvements  5. Define alternatives  6. Experiment with alternatives  7. Select best alternatives |
| --- |

Source [10]
